# Correction: A mitochondria targeted nitroreductase-sensitive self-immolative spacer as an efficient shuttle for uncharged amine-based molecules

**DOI:** 10.1039/d6sc90031c

**Published:** 2026-02-12

**Authors:** Laurane Michel, Vincent Steinmetz, Sophia Godel-Pastre, Philippe Durand, Arnaud Chevalier

**Affiliations:** a Université Paris-Saclay, CNRS, Institut de Chimie des Substances Naturelles UPR 2301 Gif-sur-Yvette 91198 France arnaud.chevalier@cnrs.fr

## Abstract

Correction for ‘A mitochondria targeted nitroreductase-sensitive self-immolative spacer as an efficient shuttle for uncharged amine-based molecules’ by Laurane Michel *et al.*, *Chem. Sci.*, 2025, **16**, 18383–18389, https://doi.org/10.1039/D5SC03665H.

The authors regret that an error occurred in the transcription of the values reported in Table 2c. The reported fluorescence QY values for compound **4-ANI** were underestimated. The corrected values are provided in Table 2c of Fig. 2.

**Fig. 2:**
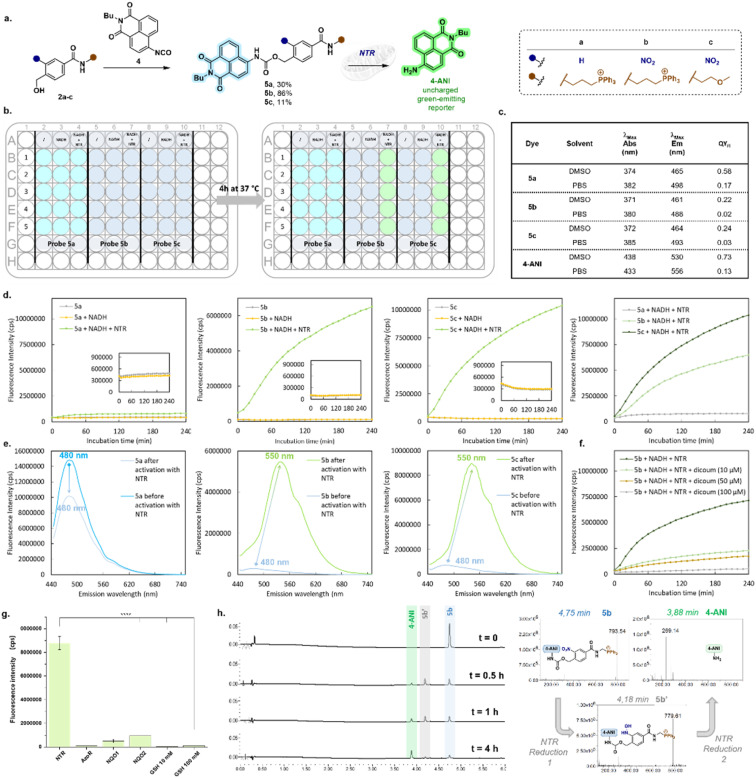
Synthesis, photophysical characterization and *in vitro* enzymatic conversion of ratiometric fluorogenic probes **5a–c**. (a) Synthesis of a **4-ANI**-based fluorogenic probe (**5a–c**) and principle of ratiometric response to NTR. (b) Schematic representation of a 96-well plate illustrating the fluorescence emission color change associated with the release of **4-ANI** following the enzymatic activation of probes **5a–c**. (c) Photophysical properties of probes **5a–c** and **4-ANI** measured in PBS buffer and DMSO at 25 °C. Relative QY_Fl_ were calculated using quinine sulfate (QY_Fl_ = 0.59 in HClO_4_ 0.1 M)^28^ for probes **5a–c** or coumarin 153 (QY_Fl_ = 0.53 in EtOH)^28^ for **4-ANI**. (d) Kinetic monitoring of NTR activation (1 µg) of probes **5a–c** (10 µM in PB buffer pH = 7.4 + NADH 500 µM). The fluorescence was recorded over time at 540 nm with excitation fixed at 435 nm. (e) Emission spectra of probes **5a–c** upon excitation at 405 nm recorded before and after incubation for 4 h with NTR in the presence of NADH (500 µM). (f) Inhibition effect of dicoumarol on **5b** activation by NTR (fluorescence was recorded over time at 540 nm with excitation fixed at 430 nm). (g) Comparison of fluorescence intensity recorded after 30 min of incubation with multiple reducing agents at 37 °C. (h) UHPLC/MS analysis of the NTR-mediated transformation of probe **5c**, giving the corresponding fluorophore **4-ANI** through the hydroxylamine intermediate **5b′** and corresponding MS spectra.

The Royal Society of Chemistry apologises for these errors and any consequent inconvenience to authors and readers.

